# National Black HIV/AIDS Awareness Day — February 7, 2020

**DOI:** 10.15585/mmwr.mm6904a1

**Published:** 2020-01-31

National Black HIV/AIDS Awareness Day (NBHAAD) is observed each year on February 7 to highlight the continuing disproportionate impact of human immunodeficiency virus (HIV) infection and acquired immunodeficiency syndrome (AIDS) on the U.S. black or African American (black) population. During 2018, blacks represented 13% of the U.S. population but accounted for 43% of all newly diagnosed HIV infections (*1*).

In February 2019, a new national initiative, Ending the HIV Epidemic: A Plan for America (EHE), was proposed. The plan calls for intensified efforts to diagnose, treat, prevent, and respond to HIV infections in the United States, with an overall goal of reducing new HIV infections by ≥90% by 2030 (*2*).

A study reported in this *MMWR* issue presents data on CDC-funded HIV testing and outcomes among blacks who were tested in jurisdictions that are the initial focus of EHE. In these jurisdictions during 2017, blacks accounted for 43.2% of CDC-funded tests and 49.1% of newly diagnosed HIV infections (*3*). CDC supports a range of efforts for reducing the risk for acquiring or transmitting HIV infection among blacks. Additional information is available at https://www.cdc.gov/hiv/group/racialethnic/africanamericans. Information about NBHAAD is available at https://www.cdc.gov/hiv/library/awareness/nbhaad.html. 

## References

[1] CDC. Diagnosis of HIV infection in the United States and dependent areas, 2018. HIV surveillance report, vol. 30. Atlanta, GA: US Department of Health and Human Services, CDC; 2019. https://www.cdc.gov/hiv/pdf/library/reports/surveillance/cdc-hiv-surveillance-report-2018-vol-30.pdf

[2] Harris NS, Johnson AS, Huang YA, Vital signs: status of human immunodeficiency virus testing, viral suppression, and HIV preexposure prophylaxis—United States, 2013–2018. MMWR Morb Mortal Wkly Rep 2019;68:1117–23. 10.15585/mmwr.mm6848e1 31805031 PMC6897528

[3] Essuon AD, Zhao H, Wang G, Collins N, Karch D, Rao S. HIV testing outcomes among blacks or African Americans—50 local U.S. jurisdictions accounting for the majority of new HIV diagnoses and seven states with disproportionate occurrences of HIV in rural areas, 2017. MMWR Morb Mortal Wkly Rep 2020;69:97–102. 31999684 10.15585/mmwr.mm6904a2 PMC7004407

